# Supplementary material for: Deep learning and transfer learning identify breast cancer survival subtypes from single-cell imaging data
Source: Commun Med (Lond). 2023 Dec 19;3:187. doi: 10.1038/s43856-023-00414-6 (PMC10730890; doi:10.1038/s43856-023-00414-6)
Supplement: Supplementary file 2 — Supplementary Information [file 43856_2023_414_MOESM2_ESM.pdf]

Deep-learning and transfer learning identify new breast cancer survival subtypes from single-cell imaging data

Authors
Shashank Yadav, Shu Zhou, Bing He,Yuheng Du, Lana X Garmire

Supplementary Information

Supplementary Tables

Supplementary Table 1: Distribution of our cohort's 259 patients corresponding to Clinical Features and clinicopathological subtypes

Clinical Features											Subtypes			
	Tumor Grade			ER Status		PR Status		HER2 Status			Luminal A	Luminal B	TNBC	HER2 Enriched
Total	I	II	III	+	-	+	-	+	-					
259	34	109	116	191	68	143	116	49	210		166	26	44	23

Supplementary Table 2: Description of patients in NMF-derived subpopulation clusters corresponding to clinical features and clinicopathological subtypes

Distribution of Patient in each Cluster Clinical Features														
**Clinical Features**											**Subtypes**			
**Cluster ID**	**No. Of Patients**	**Tumor Grade**			**ER Status**		**PR Status**		**HER2 Status**		**Luminal A**	**Luminal B**	**TN BC**	**HER2 Enriched**
		**I**	**II**	**III**	+	**-**	+	**-**	+	**-**				
**1**	23	6	9	8	14	9	12	11	7	16	14	0	2	7
**2**	65	7	35	23	59	6	45	20	13	52	48	11	4	2
**3**	51	4	15	32	27	24	19	32	12	39	21	6	18	6
**4**	76	16	41	19	71	5	55	21	8	68	65	7	3	1
**5**	23	0	3	20	9	14	5	18	6	17	8	1	9	5
6	13	0	6	7	9	4	5	8	3	10	8	1	2	2
**7**	8	1	0	7	2	6	2	6	0	8	2	0	6	0

Supplementary Figures

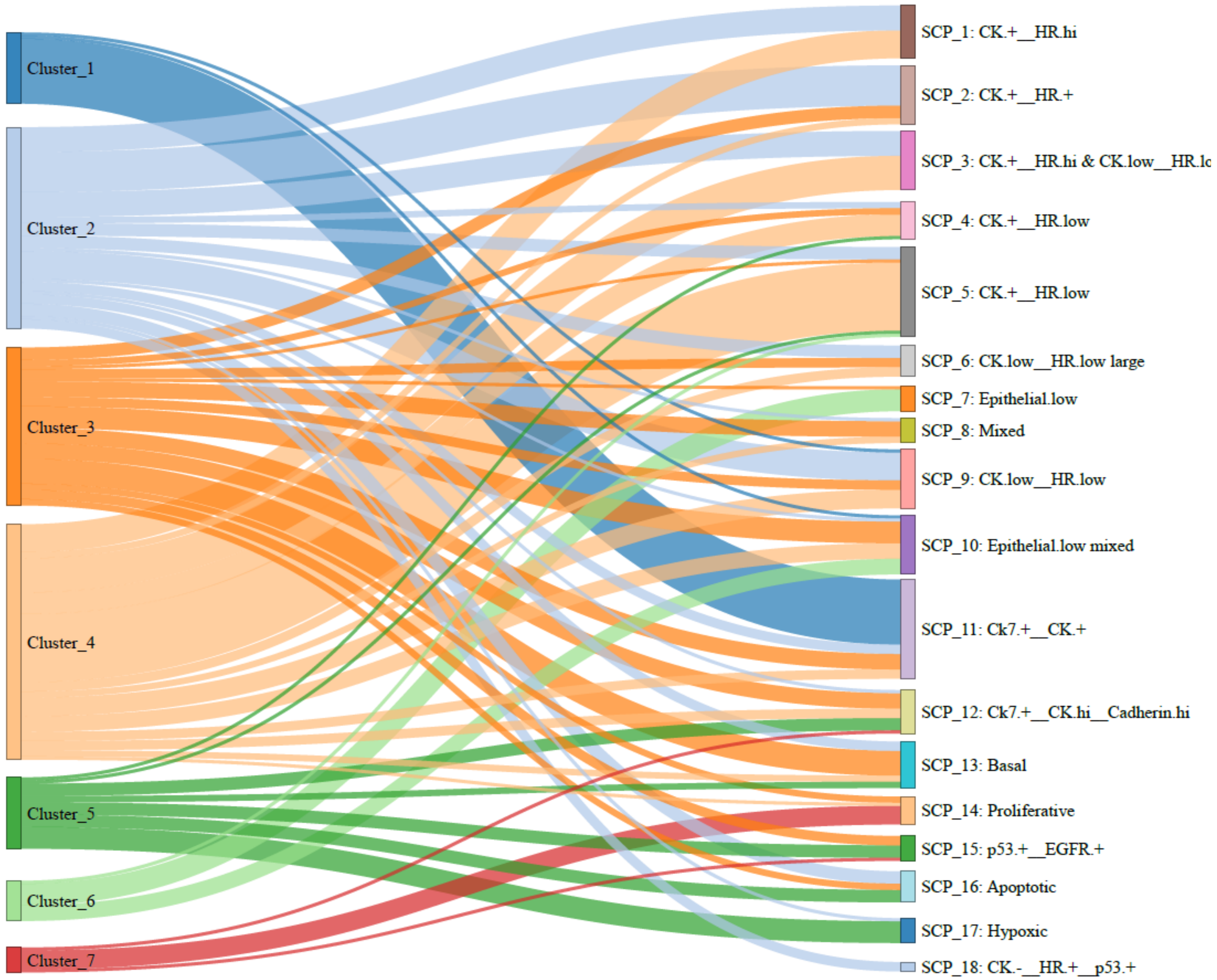

Supplementary Figure 1: Sankey plot showing the distribution of patients in the seven subpopulations vs. the original 18 single-cell pathology (SCP) subgroups.

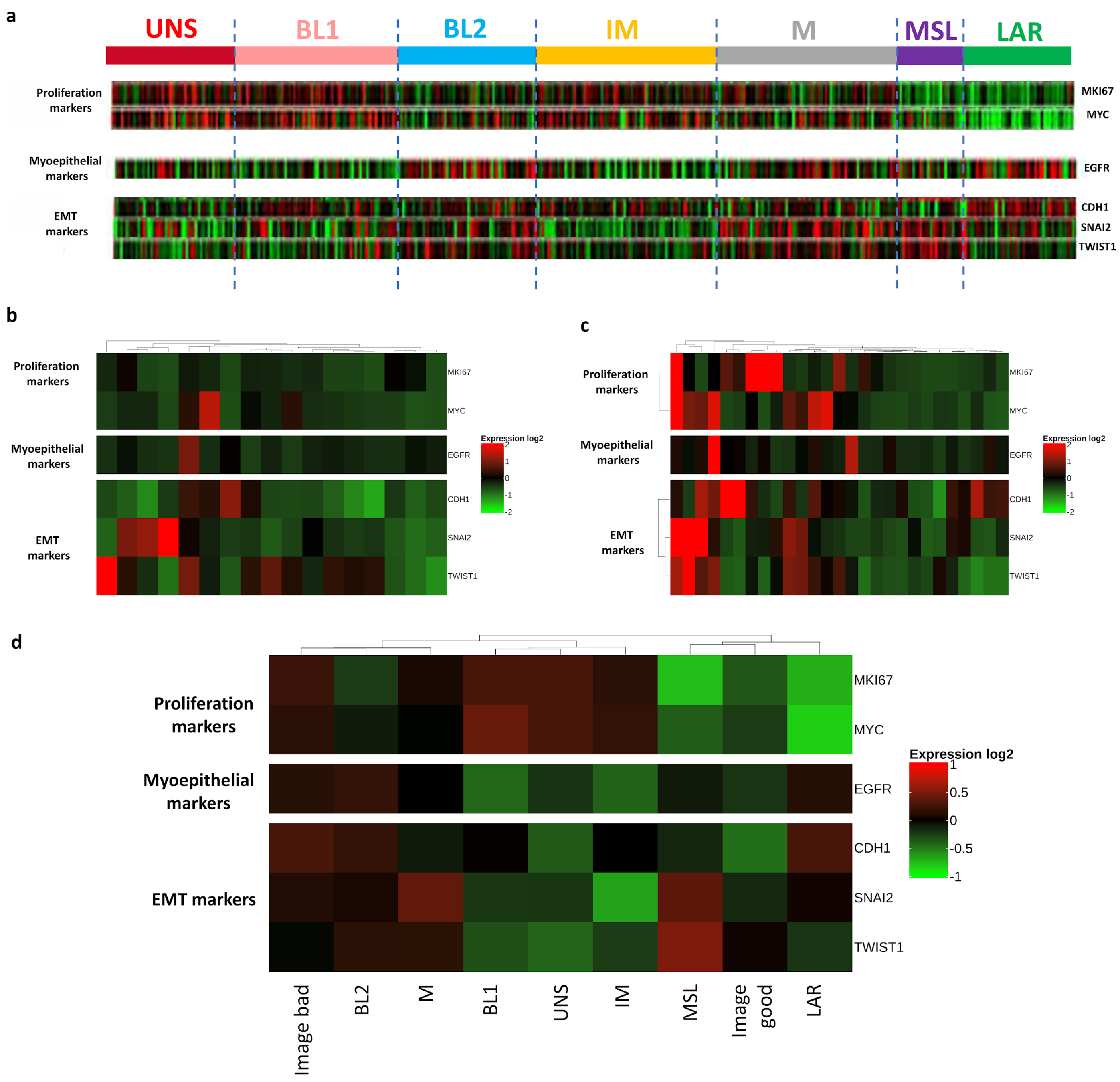

Supplementary Figure 2: Cross-check the good and bad TNBC survival subtypes with previous defined TNBC molecular subtypes. (a) [1]. **(b)** Heatmap of the expression level of the six common marker genes in the good survival TNBC subtype. **(c)** Heatmap of the expression level of the six common marker genes in the bad survival TNBC subtype. (**d**) Hierarchical clustering results on the average expression levels of the TNBC survival subtypes and the seven TNBC molecular subtypes.

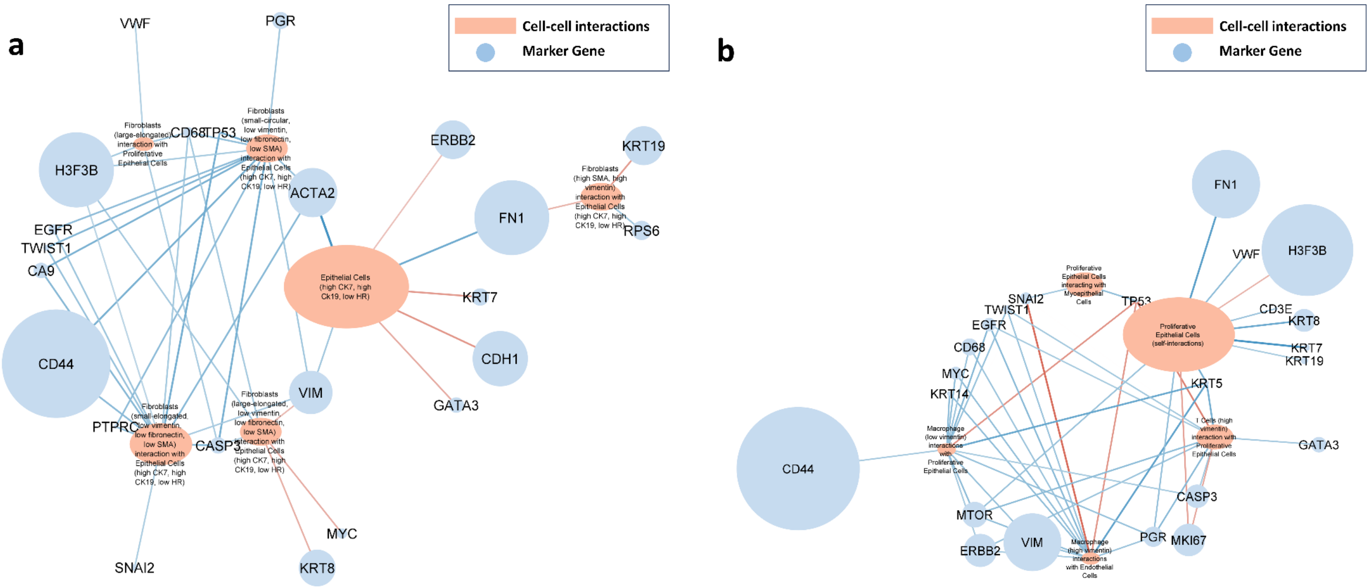

Supplementary Figure 3: Bipartite graph demonstrating the strong correlations between cell-cell interaction pairs (ellipses) and gene markers (circles) in subgroups 1 (a) and 7 (b). Ellipse: cell-cell interaction pair; circle: gene marker. Red line: positive correlations; blue lines: negative correlations. The sizes of the ellipses and circles are proportional to the average expression level of cell-cell interactions and gene expression respectively.

Supplementary References

1. Lehmann, B. D. *et al.* Identification of human triple-negative breast cancer subtypes and preclinical models for selection of targeted therapies. *J. Clin. Invest.* **121**, 2750–2767 (2011).
