# Supplementary material for: Deep learning and transfer learning identify breast cancer survival subtypes from single-cell imaging data
Source: Commun Med (Lond). 2023 Dec 19;3:187. doi: 10.1038/s43856-023-00414-6 (PMC10730890; doi:10.1038/s43856-023-00414-6)
Supplement: Supplementary file 3 — Description of Additional Supplementary Files [file 43856_2023_414_MOESM3_ESM.docx]

Description of Additional Supplementary Files

**File name:** Supplementary Data 1

**Description:** Supplementary Data 1: Annotations of the 27 Cell Phenotypes defined by (Jackson et al. 2020).

**File name:** Supplementary Data 2

**Description:** Supplementary Data 2: Significant features of the Cellular Phenotypic (CP) feature set.

**File name:** Supplementary Data 3

**Description:** Significant features of the Tumor Microenvironment Interaction (TMI) feature set.

**File name:** Supplementary Data 4

**Description:** Significant features of the Tumor Core Interaction (TCI) feature set

**File name:** Supplementary Data 5

**Description:** Results of the differential analysis to the TNBC s

**File name:** Supplementary Data 6

**Description:** Results of the differential analysis to the luminal A subtypes of single-cell imaging cohort

**File name:** Supplementary Data 7

**Description:** Results of the differential analysis to the TNBC subtypes of the TCGA data

**File name:** Supplementary Data 8

**Description:** Results of the differential analysis to the TNBC subtypes of the Metabric data

**File name:** Supplementary Data 9

**Description:** Results of the differential analysis to the luminal A subtypes of the TCGA data

**File name:** Supplementary Data 10

**Description:** Results of the differential analysis to the luminal A subtypes of the Metabric data
