# Supplementary material for: Deep learning and transfer learning identify breast cancer survival subtypes from single-cell imaging data
Source: Commun Med (Lond). 2023 Dec 19;3:187. doi: 10.1038/s43856-023-00414-6 (PMC10730890; doi:10.1038/s43856-023-00414-6)
Supplement: Supplementary file 13 — Supplementary Data 10 [file 43856_2023_414_MOESM13_ESM.pdf]

Gene Name	logFC	AveExpr	t	P.Value	adj.P.Val
KRT7	0.977340	-0.097440	3.803803	1.49E-04	4.46E-03
ACTA2	0.703218	-0.001900	3.603348	3.25E-04	4.88E-03
CDH1	-0.674130	-0.543030	-3.399420	6.95E-04	6.95E-03
VIM	0.845528	-0.193060	3.084499	2.08E-03	1.56E-02
MS4A1	-0.592870	-0.177460	-2.577770	1.00E-02	6.03E-02
VWF	0.191635	0.053709	1.934711	5.32E-02	2.66E-01
GATA3	0.096992	0.455363	1.832000	0.067168	0.287864
KRT14	0.154100	-0.197550	1.588594	0.112383	0.374411
SNAI2	0.148167	-0.055810	1.562958	0.118293	0.374411
TP53	-0.129190	0.007260	-1.488630	0.136816	0.374411
CA9	-0.096260	-0.256750	-1.486850	0.137284	0.374411
CD3E	-0.119040	-0.157230	-1.363400	0.172979	0.401474
KRT19	0.118648	0.097706	1.326799	0.184796	0.401474
KRT5	0.106327	-0.181740	1.283057	0.199689	0.401474
KRT8	0.104671	0.131979	1.280070	0.200737	0.401474
FN1	0.090496	-0.083140	0.950817	0.341865	0.622803
CD68	0.083207	-0.109750	0.891749	0.372684	0.622803
ERBB2	0.051047	-0.191510	0.889889	0.373682	0.622803
RPS6	0.081818	0.021178	0.837080	0.402693	0.635832
PGR	0.070527	0.308679	0.720813	0.471147	0.688093
EGFR	-0.046280	-0.306160	-0.691850	0.489147	0.688093
CD44	0.066715	0.038655	0.667443	0.504602	0.688093
H3F3B	-0.049260	0.133718	-0.518680	0.604066	0.787912
MTOR	0.046233	-0.057040	0.479503	0.631657	0.789572
MYC	0.036033	-0.053670	0.380451	0.703670	0.844404
CASP3	0.027063	-0.224020	0.299306	0.764752	0.850481
TWIST1	0.030437	-0.058700	0.298414	0.765433	0.850481
MKI67	-0.014180	-0.011680	-0.140220	0.888508	0.951973
PTPRC	-0.00288	-0.14908	-0.03257	0.974025	0.987457
ESR1	0.000747	0.502018	0.015724	0.987457	0.987457
